# From MASH to HCC: the role of Gas6/TAM receptors

**DOI:** 10.3389/fimmu.2024.1332818

**Published:** 2024-01-17

**Authors:** Daria Apostolo, Luciana L. Ferreira, Federica Vincenzi, Nicole Vercellino, Rosalba Minisini, Federico Latini, Barbara Ferrari, Michela E. Burlone, Mario Pirisi, Mattia Bellan

**Affiliations:** ^1^Department of Translational Medicine, Università del Piemonte Orientale, Novara, Italy; ^2^Department of Internal Medicine, Azienda Ospedaliero-Universitaria Maggiore Della Carità, Novara, Italy; ^3^Center on Autoimmune and Allergic Diseases, Università del Piemonte Orientale, Novara, Italy

**Keywords:** MASH, MASLD, HCC, NAFLD, NASH, Gas6, TAM receptors, liver

## Abstract

Metabolic dysfunction-associated steatohepatitis (MASH) is the replacement term for what used to be called nonalcoholic steatohepatitis (NASH). It is characterized by inflammation and injury of the liver in the presence of cardiometabolic risk factors and may eventually result in the development of hepatocellular carcinoma (HCC), the most common form of primary liver cancer. Several pathogenic mechanisms are involved in the transition from MASH to HCC, encompassing metabolic injury, inflammation, immune dysregulation and fibrosis. In this context, Gas6 (Growth Arrest-Specific 6) and TAM (Tyro3, Axl, and MerTK) receptors may play important roles. The Gas6/TAM family is involved in the modulation of inflammation, lipid metabolism, fibrosis, tumor progression and metastasis, processes which play an important role in the pathophysiology of acute and chronic liver diseases. In this review, we discuss MASH-associated HCC and the potential involvement of the Gas6/TAM system in disease development and progression. In addition, since therapeutic strategies for MASH and HCC are limited, we also speculate regarding possible future treatments involving the targeting of Gas6 or TAM receptors.

## 1 Introduction

With Non-Alcoholic Fatty Liver Disease (NAFLD) we referred to a broad spectrum of chronic liver diseases, characterized by the abnormal accumulation of triglycerides in the hepatocytes. The nomenclature regarding this condition has been recently revised by the American Association for the Study of Liver Disease (AASLD) and by the European Association for the Study of the Liver (EASL) as Metabolic dysfunction-Associated Steatotic Liver Disease (MASLD) (1). MASLD includes patients with hepatic steatosis and at least one of the five cardiometabolic risk factors: Body Mass Index (BMI) ≥ 25 kg/m^2^, elevated glucose levels or Type 2 Diabetes (T2D), blood pressure ≥ 130/85 mmHg, plasma triglycerides ≥ 1.70 mmol/L or plasma High Density Lipoprotein (HDL)-cholesterol ≤ 1.0 mmol/L. In addition, a new separated subcategory, named “MetALD”, has been created to classify patients with coexisting metabolic dysfunctions and weekly alcohol intake ranging between 140-350 grams for female and 210-420 grams for male (2). By the last three decades, MASLD has become a common disorder affecting approximately 30% of the population worldwide, with a particularly high prevalence among elderly (3, 4). MASLD encompasses two clinic-pathological entities including simple steatosis, which usually ends up in a benign non-progressive clinical course, and steatohepatitis (NASH), which could potentially evolve into cirrhosis and hepatocellular carcinoma (HCC) (5).

In coherence with the new nomenclature, NASH has been renamed Metabolic Associated Steatohepatitis (MASH). MASH is characterized by lobar inflammation, hepatocyte injury and apoptosis, elevated local and systemic cytokines, activation of Hepatic Stellate Cells (HSCs) and expansion of Liver Progenitor Cells (LPCs) in periportal areas (6, 7). The gold standard for MASH diagnosis is liver biopsy, followed by histological evaluation that allows the identification of key findings, including cell ballooning and Mallory-Denk bodies, in addition to steatosis (accumulation of fat) and inflammation with or without fibrosis (8, 9).

The pathogenesis of MASH is mediated by the accumulation of free fatty acids in the liver, which enhances oxidative stress, and uncontrolled lipid oxidation, leading to the production of toxic lipids. These harmful metabolites damage hepatocytes and cause lipoapoptosis, a primary cause of persistent inflammation. Over time, the increased lipotoxicity and the inflammatory environment can lead to the progression of fibrosis, with accumulation of extracellular matrix and formation of scars in the liver parenchyma (10, 11).

MASH is a key step from the progression of MASLD to cirrhosis, which may be further complicated by portal hypertension, ascites, hepatic encephalopathy and HCC. However, there have been reported some cases of MASLD-related HCC even in the absence of advance fibrosis (12). HCC is the sixth most common cancer and the third-leading cause of cancer related mortality worldwide (13). Hepatocarcinogenesis is a complex multistep process associated with the transition from hepatocyte damage, fibrosis deposition and portal vascularization, towards a context of high-capacity proliferative cells with invasive and metastatic potential (14). Major risk factors for HCC include HBV or HCV related hepatitis, aflatoxins, chronic alcohol consumption, metabolic dysfunctions such as obesity and diabetes, hereditary hemochromatosis and immune related conditions; in industrialized countries, however, the strongest predictor of HCC is cirrhosis of any etiology (15). MASLD/MASH-derived HCC exhibits distinctive traits compared with HCC from different etiologies (16). It is currently known that patients with MASLD/MASH-derived HCC have lower survival rates compared to patients with viral hepatitis HCC (17). While the mechanisms behind the development of HCC from viral infection are widely known (18, 19), the pathogenetic mechanisms underlying MASH-related HCC are not fully understood. However, it is known that tumorigenesis involves environmental factors along with mutations of genes implicated in cell proliferation, apoptosis, as well as genetic variants that predispose to liver fat accumulation. Among them, the variant (rs738409 c.444 C>G, p.I148M) in patatin-like phospholipase domain-containing protein 3 (*PNPLA3*), which influences the activity of the PNPLA3 enzyme resulting in increased accumulation of fat in the liver, has been associated with predisposition to fatty liver disease and HCC (20, 21). Moreover, it has been reported that PNPLA3 may have a potential role in retinol metabolism and HSCs biology, thus contributing to fibrosis (22). Furthermore, loss of function of transmembrane 6 superfamily member 2 (*TM6SF2*) gene may contribute to the pathogenesis of MASH and HCC. Indeed, patients with TM6SF2 genetic variant (rs58542926 c.449 C>T, p.E167K) exhibit increased levels of liver triglycerides and reduced release of very low density lipoproteins from hepatocytes (23, 24). Along with metabolic-related variants, other genes that regulate proliferation, cell cycle and DNA repair are involved in the pathogenesis of HCC: a) mutation in Telomerase Reverse Transcriptase (*TERT*) gene promoter occurs in early carcinogenesis process and avoids the replicative senescence; b) Tumor protein P53 (*TP53*) gene mutation impairs DNA repair and damage response and is linked to the HBV-related HCC; c) Catenin Beta 1 (*CTNNB1*) gene mutation leads to deregulated Wnt/β-catenin signaling; d) Retinoblastoma protein 1 (*Rb1*) and Cyclin Dependent Kinase Inhibitor 2A (*CDKN2A*) genes mutations affect the transition from G1 to S phase of the cell cycle; e) Fibroblast Growth Factors 3, 4, 19/Cyclin D 1 (*FGF3, FGF4, FGF19/CCND1*) amplification, TP53 and CDKN2A alterations result in aggressive tumors in late stages; f) Activin A Receptor Type 2A (*ACVR2A*) gene downregulation induces cell proliferation and migration, resulting in poor outcomes (25, 26).

In HCC, growth factor receptors (e.g., EGFR, epidermal growth factor receptor; FGFR, fibroblast growth factor receptor; IGFR, insulin-like growth factor receptor; TGFA, transforming growth factor alpha; VEGFR, vascular endothelial growth factor receptor), cytoplasmic intermediates (e.g., PI3K-AKT-mTOR, RAF/ERK/MAPK) and key pathways in cell differentiation (e.g., Hippo, Hedgehog, Notch, JAK/STAT, Wnt/β-catenin) represent the most common altered mechanisms (27).

## 2 Gas6 and TAM receptors

### 2.1 Structure and role in cell physiology

TAM receptors belong to the family of receptor tyrosine kinases (RTKs) and include three membrane receptors: Tyro3, Axl and myeloid-epithelial-reproductive tyrosine kinase (MerTK) (28). They were identified in 1991 and afterwards cloned as full length RTKs: Tyro3 from the central nervous system (CNS) (29), Axl from chronic myelogenous leukemia (30), and MerTK from B-lymphoblastoid cells (31). All share a similar structure which consists of two immunoglobulin-like (IgL) repeats, two fibronectin type III (FNIII) domains connected to a single-pass transmembrane domain and an intracellular cytoplasmic tyrosine kinase domain (TKD) (32).

These receptors differ in cell and tissue expression (33). Indeed, Tyro3 is widely present in CNS, but it is also expressed in Sertoli cells, platelets, mature osteoclasts and retina (34–38). Axl is expressed in a wide variety of tissues and organs including brain, monocytes, macrophages, platelets, endothelial cells and dendritic cells (39, 40). MerTK has been mainly described in retinal pigment epithelium, peripheral blood and bone marrow mononuclear cells, monocytes, and macrophages (36, 41).

The TAM receptors are activated upon the binding of their extracellular ligands, protein S (Pros1) and growth arrest-specific protein 6 (Gas6), leading to receptor dimerization and activation of the tyrosine kinase domain (42, 43). In addition, tubby-like protein and galectin-3 represent other described ligands of TAM receptors (44, 45). Pros1 and Gas6 are two homologous vitamin K-dependent proteins; the former acts as a ligand for Tyro3 and MerTK, the latter binds all three TAMs with higher affinity for Axl (46). Pros1 and Gas6 have a similar structure composed by an N-terminal γ-carboxyglutamate (Gla)-rich domain, a loop region, four epidermal growth factor (EGF)-like domains, and a C-terminal sex hormone-binding globulin (SHBG)-like domain containing two globular laminin G-like domains that are able to mediate the binding between ligand and receptor (47). Moreover, metalloproteases are capable of cleaving the extracellular domains of TAMs with the consequent inactivation of these receptors and the production of their soluble circulating forms (sTyro3, sAxl, sMerTK) (48, 49). The activation of TAM receptors is linked to several signal transduction pathways including phosphoinositide 3 kinase (PI3K)/AKT, growth factor receptor-bound protein 2 (Grb2), mitogen-activated protein (MAP) kinase, nuclear factor κ-light-chain-enhancer of activated B cells (NF-κB), signal transducer and activator of transcription protein (STAT), phospholipase c-γ, Raf-1 and extracellular-signal-regulated kinase (ERK) (50–52).

The TAM family is involved in several physiological processes, including modulation of inflammation, platelet aggregation, smooth muscle homeostasis, as well as regulation of efferocytosis (53–55). In addition, it has been described that this system also contributes to the stimulation of cell growth and proliferation, migration and immune responses (56). In turn, dysregulation of TAM signaling has been implicated in various autoimmune disorders such as rheumatoid arthritis and systemic lupus erythematosus, and degenerative diseases, including Alzheimer’s disease and multiple sclerosis (57–62).

### 2.2 Gas6/TAM in inflammation and carcinogenesis

Inflammation is a dynamic physiological response to tissue damage or harmful stimuli, characterized by the release of several inflammatory mediators that coordinate cellular defense mechanisms and tissue repair. It is also marked by vascular dilation and enhanced capillary permeability, facilitating the recruitment of leukocytes to the damaged tissue. The inflammatory process is essential in the maintenance of tissue homeostasis and is usually a temporary and well-orchestrated response. However, under certain circumstances, the acute inflammatory process may turn into an excessive and prolonged response, with consequent deleterious effects on the healthy tissues and associated with several diseases, including autoimmune disease, metabolic disorders, heart disease and cancer (63). Therefore, the balance between the beneficial and detrimental effects of inflammation can be delicate and requires fine-tuned maintenance mechanisms. The role of TAM signaling in the modulation of the inflammatory process and development of immune response has been highlighted over the past years. TAM receptors are believed to be involved in the suppression of inflammation through a complex negative feedback mechanism. In dendritic cells, it was proposed that activated TAM receptors can associate with IFNAR-STAT1 complex and induce transcription of SOCS1 (suppressor of cytokine signaling-1) and SOCS3. These two proteins inhibit toll-like receptors (TLRs) and TLR-induced cytokine-receptor cascades, switching off the inflammatory response. In turn, the activation of TLR leads to a burst of cytokines resulting in an IFNAR/STAT1-dependent upregulation of the TAM system (64, 65). Inhibition or ablation of TAM receptors may lead to inflammation and autoimmunity. This is particularly evident in TAM-deficient mice that eventually develop autoimmune disease, probably due to the dysregulation of the inflammatory process and the loss of phagocytic response to apoptotic cells (41, 66). Macrophages are important during inflammation, due to their role in the engulfment of apoptotic cells that need to be efficiently cleared from the body. In macrophages, this phagocytic process occurs mostly via MerTK and Axl receptors (67, 68). Genetically modified mice with a cytoplasmic truncation of MerTK show defective macrophages with impaired capacity in the clearance of apoptotic cells (66). Clinically, elevated levels of Gas6 and TAM receptors have been reported in several inflammatory diseases (69–72).

Chronic inflammation has also been linked to increased cancer risk and more advanced tumor stage (73, 74). TAM receptors and Gas6 have been studied in different types of cancers and Gas6/TAM-related signaling pathways are associated with the activation of standard proliferative pathways (including MEK/ERK and PI3K/AKT), implicated in cell proliferation, growth and survival (37, 75). The overexpression of MerTK and Tyro3, but mostly Axl, has been reported in several solid and hematological tumors and is usually associated with increased metastatic risk and drug resistance, as well as overall worst prognosis (76–79). However, the exact mechanisms that lead to the abnormal expression and activation of TAM receptors in cancer remain unclear. One possible mechanism relies on the interaction and crosstalk between hypoxia-inducible factor-1 alpha (HIF-1α) and Axl. While HIF-1α, often found upregulated in cancer, has the capacity to regulate the expression of Axl, Axl also seems to be important in the transcription and stability of HIF-1α. In fact, during hypoxia, the inhibition of Axl suppresses hypoxia-induced epithelial-to-mesenchymal transition (EMT) and invasion (80). DNA methylation, microRNA, long non coding RNA (lncRNA) and other transcriptional activators have also been pointed out as regulating mechanisms of Axl expression and mRNA stability (81–84). Accordingly, Bemcentinib (R428), a selective Axl inhibitor, is currently in phase I/II clinical trials of Acute Myeloid Leukemia (AML), glioblastoma and malignant mesothelioma and has completed phase II clinical trials in patients with non-small cell lung cancer (NCT02424617) or metastatic pancreatic cancer (NCT03649321). The soluble form of the receptors was also suggested as a marker for the prediction of prognosis in cancers of different origin. For example, increased sAxl levels were observed in late-stage melanoma patients compared to patients at an earlier stage (85), whereas lower levels of sAxl and Gas6 in serum was associated with longer survival in renal cell carcinoma patients (86). Similarly, both in pancreatic ductal adenocarcinoma (87) and in high-grade serous carcinoma (88) patients, sAxl was significantly increased.

### 2.3 Gas6/TAM in liver disease

Several studies have shed light on the involvement of Gas6/TAM system in liver diseases. Gas6 and Axl signaling has a particularly relevant role in the development of liver fibrosis, by promoting HSC activation, a key step in fibrotic tissue formation. Indeed, Gas6/Axl system activates PI3K/AKT signaling and stimulates NF-kB p65 translocation to the nucleus inducing an anti-apoptotic response against HSCs. This sustains HSCs survival and proliferation, promoting the progression from steatosis to fibrosis (89–91). Under normal conditions, Gas6 and Axl are expressed by Kupffer cells (KCs) and liver sinusoidal endothelial cells. However, in case of liver injury, fibrosis, and malignant transformation, they are upregulated, promoting cell survival, fibrosis, and angiogenesis (91–93). As cirrhosis progresses, the expression of Axl by liver macrophages decreases; this might be the consequence of a down-regulation mediated by Gas6, which is conversely upregulated by HSCs, suggesting that Axl may have a role in regulating hepatic immune homeostasis (94). Axl expression is particularly concentrated in HSCs reflecting its profibrogenic role (89). Indeed, Axl depletion in LX2 cells by small interfering RNA (siRNA) transfection has been shown to induce a significant reduction in Alpha-Smooth Muscle Actin (α-SMA) levels, underpinning the relevance of Gas6/Axl axis for full HSC activation and proliferation *in vitro* (91).

The current evidence suggests that TAM receptor signaling plays a divergent role in liver diseases. While the system might be beneficial in acute liver injury, it may be deleterious in chronic liver diseases. Indeed, the activation of TAM receptors can modulate immune responses, promote tissue repair, and reduce inflammation limiting the extent of damage and contributing to the recovery process. In *in vivo* experiments, Gas6-deficient mice fed with choline-deplete ethionine (CDE)-supplemented diet to develop steatohepatitis showed a reduction in HSCs activation and expression of transforming growth factor beta (TGFβ) along with a delayed onset of necroinflammation and steatosis compared to wild-type mice (95). Furthermore, Gas6 knockout (Gas6^-/-^) mice fed with CDE diet or treated with carbon tetrachloride (CCl_4_) have shown an improvement of steatohepatitis and fibrosis compared to control mice. This mechanism has been related to the inhibition of the inflammatory response that regulates lipid metabolism and myofibroblast activation (96). Conversely, when Gas6-deficient mice are exposed to an acute liver damage, such as after the injection of CCl_4_, they display a delayed repair of liver necrotic areas in comparison with wild-type mice (97). Additionally, it has been demonstrated that Gas6, released during murine hepatic ischemia/reperfusion damage, acts as survival factor protecting the hepatocytes and reducing the production of inflammatory cytokines (98).

It has been observed that autophagy in macrophages is induced by Gas6/Axl signaling through MAPK14, thanks to the tyrosine 815 and 860 residues contained in the cytoplasmic domain of Axl. Moreover, the study showed that autophagy induced by Axl is able to decrease liver injury by inhibiting NLR Family Pyrin Domain Containing 3 (NLRP3) inflammasome activation in murine acute liver injury models (99). In mice treated with CCl_4_, the intrahepatic expression of MerTK was upregulated more than 6-fold with respect to control mice, supporting the profibrogenic role of MerTK driven by inflammatory and metabolic pathways (100).

In contrast, chronic activation of TAM receptors could potentially lead to sustained immune suppression, impaired phagocytic clearance, and increased tissue fibrosis exacerbating the progression of chronic liver diseases. Indeed, Dengler et.al, analysing sAxl serum levels in MASLD patients and healthy controls, noted that median concentrations of sAxl were comparable, suggesting that in the absence of advanced fibrosis/cirrhosis no increased sAxl levels were registered. On the contrary, large cohorts of patients with F3/F4 fibrosis display elevated levels of sAxl. The upregulation of sAxl in HCC contributes to tumor progression; indeed, serum concentrations of sAxl in patients were significantly higher at early and late stages of HCC (82.57 ng/mL and 114.50 ng/mL, respectively) compared to healthy controls (40.15 ng/mL) (101). Conversely, Tutusaus et al. reported that elevated levels of sAxl were found also in early stages of MASLD, when liver fibrosis was still absent (102).

Recently, the potential diagnostic value of Gas6 and its albumin ratio was evaluated in patients with biopsy-proven chronic liver diseases, revealing that Gas6/albumin ratio possesses high accuracy in detecting advanced fibrosis and cirrhosis and predicts the severity of liver disease (103).

### 2.4 Gas6/TAM in MASH and fibrosis

In MASH, liver inflammation triggers the activation of KCs, as well as cells of the immune system that infiltrate the liver from the bloodstream, leading to fibrosis progression (104). KCs and monocyte-derived macrophages are indeed essential for the maintenance the immune function and serve as the first-line defense against pathogens. In the context of liver inflammation, M1 macrophages contribute to hepatic steatosis, inflammatory cells recruitment and fibrosis activation, while M2 macrophages have anti-inflammatory and reparative functions. Thus, it has been demonstrated that M1/M2 ratio gradually increases during MASLD progression (104, 105). After the development of hepatic steatosis, KCs secrete chemotactic substances facilitating the infiltration of monocytes. The latter are further capable of secreting pro-inflammatory cytokines and promoting the progression to fibrosis (106). Several liver diseases, including MASH, alcoholic cirrhosis and fibrosis, viral hepatitis, and cholestasis can be exacerbated and worsened by a defective activity of KCs. Thus, KCs are tightly associated with the occurrence and development of MASH (104).

Recently, the Gas6/TAM system has been evaluated as a potential therapeutic target both *in vitro* and *in vivo*. Indeed, the exposure of HSCs to medium conditioned by MerTK-expressing THP-1 cells ended up in the upregulation of expression of several genes involved in fibrogenesis, including transforming growth factor-β1 (TGFβ1), actin alpha 2 smooth muscle (ACTA2), tissue inhibitor of metalloproteinase 1 (TIMP1), vascular endothelial growth factor-A (VEGF-A), collagen type I alpha 1 chain (COL1A1), matrix metalloproteinase 2 (MMP2) and MMP9. These effects were inhibited using the specific MerTK inhibitor UNC569, highlighting the implication of this receptor in the pathogenesis of liver fibrosis (107). Similarly, it has been reported that MerTK signaling resulted in the activation of ERK-TGFβ1 pathway that stimulates HSCs and promotes liver fibrosis in MASH (108). In HSCs derived from a murine model of MASH, a reduction of fibrosis was observed following the blockage of Gas6-induced TAM activation through RU-301 or the use of all-trans retinoic acid (ATRA). The latter induces MerTK cleavage in macrophages by activating A Disintegrin and Metalloprotease 17 (ADAM17). Accordingly, mice with a MerTK receptor that was resistant to ADAM17 cleavage exhibited increased liver fibrosis. The profibrogenic role of MerTK was also demonstrated in Gas6-stimulated HSCs, which induced procollagen I gene expression. These effects were counteracted by MerTK inhibition. Moreover, a reduction in procollagen I gene expression was also obtained after MerTK silencing. The authors also evaluated the impact of MerTK rs4374383 variants in a cohort of 533 patients who underwent liver biopsy for suspected MASH.

**Figure 1:**
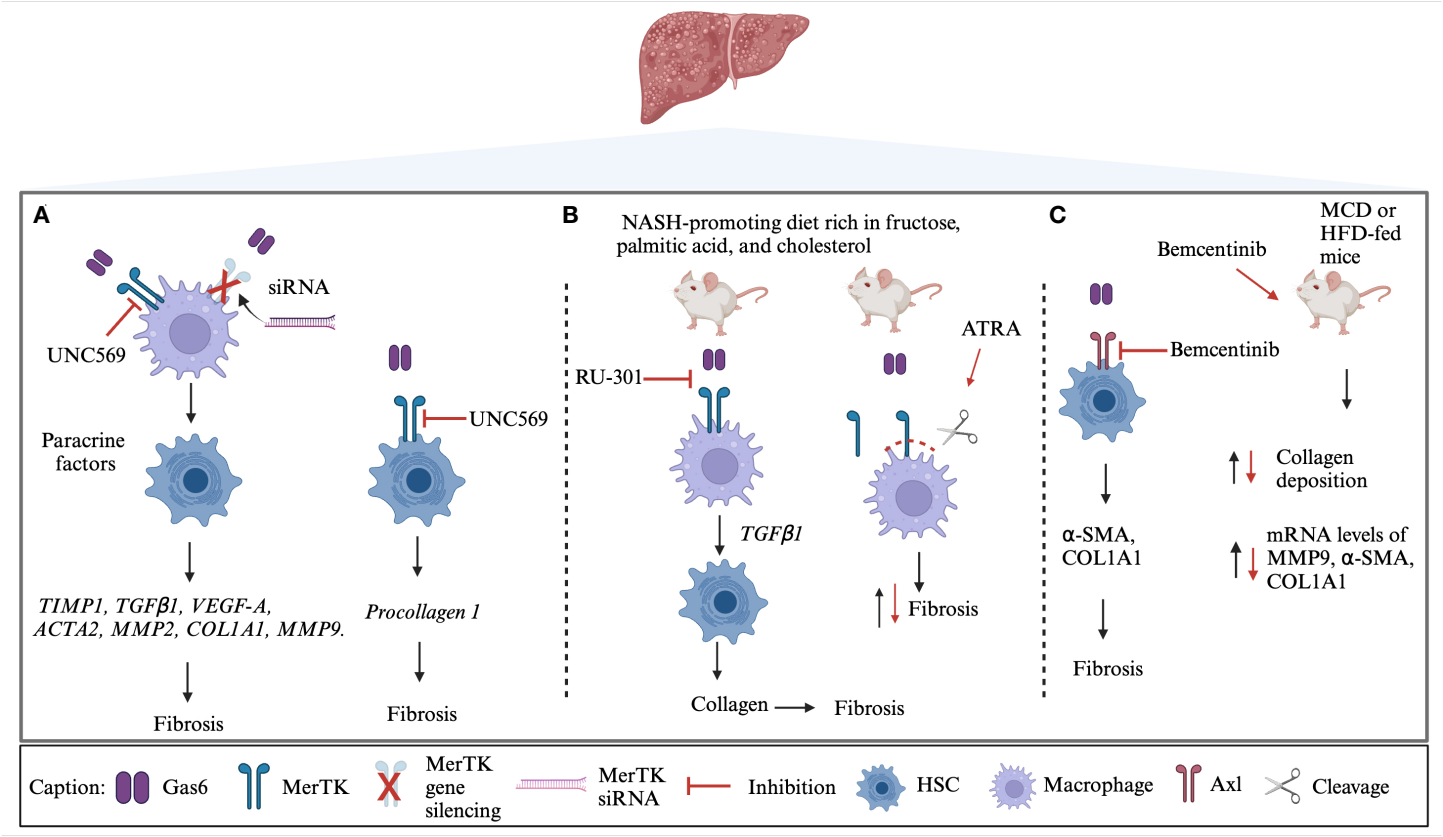
TAM targeted strategies in MASH. **(A)** MerTK on macrophages promotes the profibrogenic cross-talk with HSCs through soluble mediators. UNC569, a specific MerTK inhibitor that acts both on macrophages and HSCs, and MerTK silencing are able to inhibit the downstream activation of several genes involved in fibrosis development (100, 107). **(B)** In MASH mice-derived macrophages, the blockage of Gas6-induced TAM activation by RU-301 or MerTK cleavage by ATRA decreases fibrosis (108). **(C)** Bemcentinib (R428) treatment *in vitro* resulted in reduced fibrosis. The administration of Bemcentinib in mice induced a decrease of collagen deposition and expression of profibrogenic genes (102). Black lines represent the physiological process while red lines represent the effects of TAM targeted strategies. siRNA, small-interfering RNA; TIMP1, Tissue Inhibitor of MetalloProteinase 1; *TGFβ1*, Transforming Growth Factor- beta 1*; VEGF-A*, Vascular Endothelial Growth Factor-A*; ACTA2*, Actin Alpha 2 smooth muscle*; MMP2*, Matrix MetalloProteinase 2*; COL1A1*, Collagen type I alpha 1 chain; *MMP9*, Matrix MetalloProteinase 9; HFD, High Fatty Diet; ATRA, All-Trans Retinoic Acid; α-SMA, alpha-Smooth Muscle Actin; MCD, Methionine/Choline deficient Diet. Created with BioRender.com.

Fibrosis stage F2-F4 was observed in 19% and 30% of patients MerTK AA homozygotes and MerTK GG/GA, respectively. The rs4374383 AA genotype was found to be protective and associated with lower MerTK hepatic expression (100). Gas6 signaling via MerTK/AKT/STAT3 axis has emerged as a mechanism of hepato-protection against lipotoxicity which is known to contribute to the liver damage found in MASH. In particular, primary mouse hepatocytes (PMHs) treated with palmitic acid (PA), reveal Gas6 role in decreasing palmitic-induced PMH cell death (102). Further, Axl inhibition prevented profibrotic and proinflammatory effects and induced Gas6 upregulation as a possible compensatory mechanism, potentially preserving other liver protecting functions of the Gas6 system. In mice models, both Axl deficiency (Axl^-/-^ mice) or Axl pharmacological inhibition with Bemcentinib, were able to decrease experimental liver fibrosis after chronic administration of CCl_4_ (91). In addition, the treatment with Bemcentinib in a MASH model was able to decrease cytokine production in LPS-treated KCs. Similarly, transcriptome profiling revealed a reduction in the expression of genes involved in fibrosis and inflammation in HFD-fed mice after the administration of Bemcentinib. In this experimental model, MerTK^-/-^ mice showed an enhanced MASH phenotype and, on the contrary, Axl^-/-^ mice were partially protected (102). At the light of the current literature, the potential therapeutic strategies aimed to target Gas6/TAM system *in vitro* and *in vivo* are summarized in **Figure 1**.

### 2.5 Gas6/TAM in HCC

**Figure 2:**
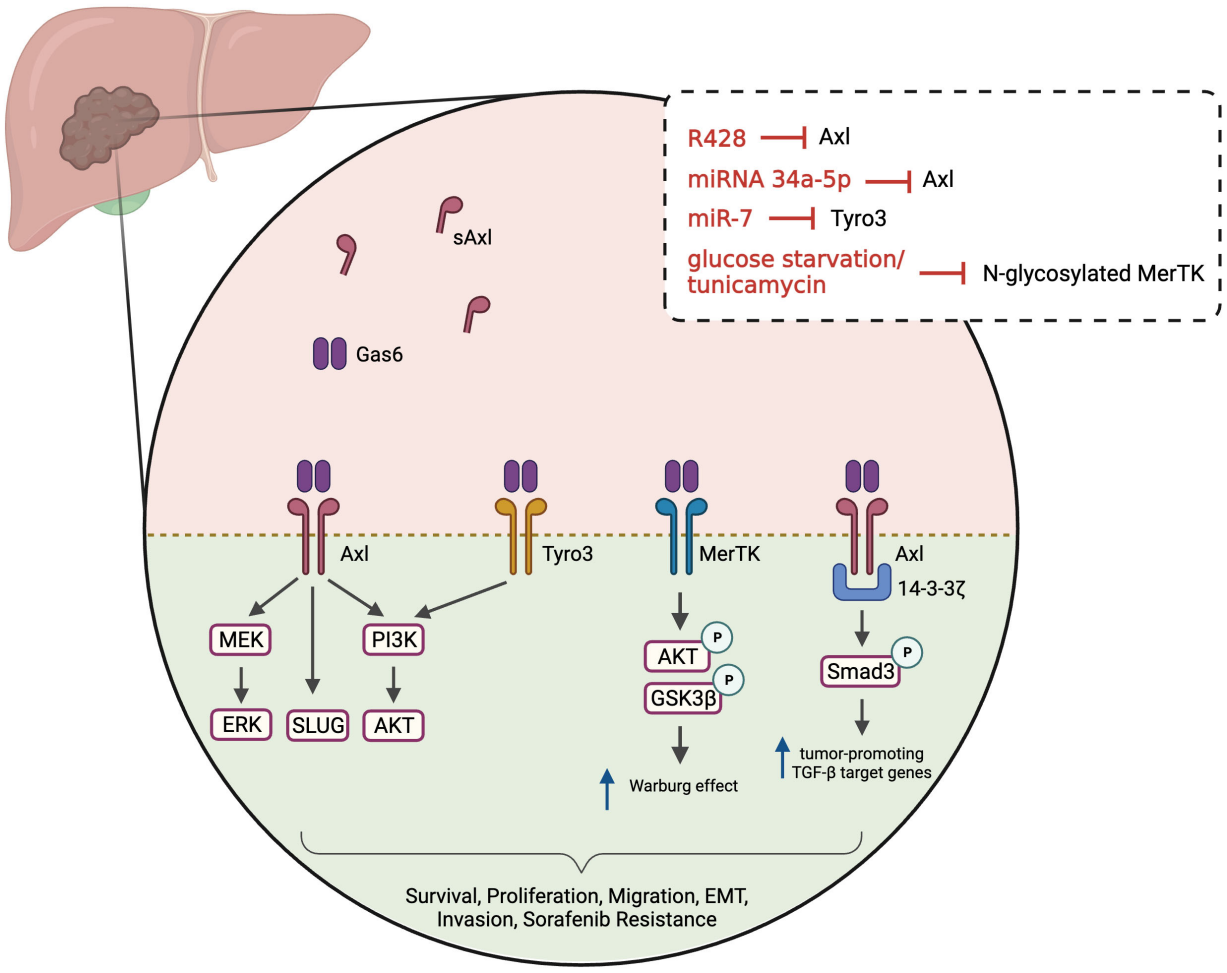
Gas6/TAM-regulated pathways in HCC. Ligand binding to the TAM receptors (Axl, MerTK and Tyro3) activates various downstream signaling pathways, including PI3K/AKT, MEK/ERK and Slug. Axl may interact with 14-3-3ζ leading Smad3 phosphorylation and up-regulation of tumor-promoting target genes of TGFβ (PAI1, MMP9, Snail, TGFβ1) in mesenchymal cells. MerTK regulates AKT/GSK3β signaling and modulates cellular bioenergetics. These pathways will lead to multiple phenotypes, including tumor cells survival, proliferation, invasion, increased migration, EMT and resistance to sorafenib. Soluble Axl is increased in HCC patients and is a candidate serum marker for diagnosing/staging HCC. Several treatments or culture media conditions have been used to inhibit and study TAM receptors, namely R428 (pharmacological inhibition of Axl), microRNAs (miRNA 34a-5p and miR-7) and modulators/blockers of MerTK glycosylation. Created with BioRender.com.

HCC remains one of the leading causes of cancer-related death, with a median survival of 6-10 months (109). The involvement of TAM receptors in the progression of HCC, either in the regulation of cell proliferation, survival and invasion or in chemoresistance, has been highlighted in recent years. In HCC, Axl is involved in the modulation of PI3K/AKT, ERK/MAPK and TGFβ signaling pathways, resulting in enhanced tumor growth and metastatic dissemination (**Figure 2**) (110, 111). Increased stimulation of HCC cells with Gas6 leads to dose-dependent Axl activation and enhanced cell invasion (112). On the other hand, Axl inhibition, by transient Axl-specific short hairpin RNA (shRNA) and by pharmacological treatment with R428, proved not only to induce phenotypic alterations in Axl-overexpressing cells but also impairs colony formation and inhibit tumor invasion (112–114). In a comprehensive study from Pinato et al., the authors evaluated the expression of Axl in a panel of immortalized HCC cell lines and investigated the relationship between Axl expression and EMT-related genes (114). They observed Axl overexpression in 13 of the 28 studied cell lines and confirmed that Axl overexpression correlated positively with Slug and Vimentin expression and negatively with E-Cadherin, supporting Axl association with EMT. Others suggested that MAPK pathway might be the major activated pathway for the induction of Slug expression by Gas6/Axl (112). In line with these results, Axl was investigated in mesenchymal and epithelial HCC cell lines, and increased Axl expression was observed in the former cells, while strongly correlating with an enhanced migratory phenotype (110). Furthermore, Axl was found to interact with 14-3-3ζ, which seems to be essential for the modulation of TGFβ signaling. According to the authors, Axl/14-3-3ζ signaling mediates the downstream phosphorylation of Smad3 linker region (Smad3L) which in turn is considered responsible for the up-regulation of tumor-promoting TGFβ target genes (**Figure 2**). Other proteins described as being regulated by the Gas6/Axl pathway in HCC include Cyr61 (115) and CXCL5 (116).

MerTK levels were also investigated in HCC patients and in preclinical HCC models (117). Whereas increased MerTK protein levels were detected in tumor tissue compared with adjacent normal tissue, no differences were found in terms of MerTK mRNA levels. Evidence showed that MerTK is N-glycosylated in HCC cells and that this post-translational modification stabilizes MerTK and promotes oncogenic transformation. Similar to what was observed with Axl, MerTK downregulation suppresses HCC cell growth *in vitro* and *in vivo.* MerTK ablation and overexpression were found to regulate the phosphorylation of Akt and GSK3β, as well as the cell bioenergetics. In fact, MerTK seems to regulate the expression of glycolytic enzymes, facilitating the Warburg effect (**Figure 2**).

The role of Tyro3, the least characterized member of the TAM family, was investigated in a cohort of 55 HCC patients (118). Tumor tissues and matched adjacent normal tissue were obtained during resection and Tyro3 gene expression was evaluated. Approximately 42% of patients exhibited Tyro3 overexpression in tumor tissues (>2-fold) and high Tyro3 expression was significantly associated with higher levels of alpha-fetoprotein (AFP). These results led others to investigate the role of Tyro3 in HCC and in the development of sorafenib resistance (119). Sorafenib is a first-line therapy in advanced HCC patients, able to lengthen the median survival time (120). Mechanistically, it works as a multi-target tyrosine kinase inhibitor, and exhibits anti-angiogenic and anti-proliferative effects. However, sorafenib treatment is associated with a variety of adverse side effects and is often followed by drug resistance (121). Acquired resistance is commonly developed when alternative signaling pathways are activated during kinase-targeted therapy to maintain survival and cell proliferation. Kabir and colleagues identified a microRNA (miR-7) capable of inhibiting the proliferation, migration and invasion of HCC cells *in vitro* and *in vivo* (119). With further investigation, they confirmed that Tyro3 is a miR-7 target gene and that Tyro3 plays an important pro-proliferative and pro-invasive role in HCC cells, through the regulation of the PI3K/AKT pathway. Since amplified expression of Tyro3 was observed in sorafenib-resistant HCC cells, it was also proposed that combining miR-7 with the inhibition of Tyro3 might represent a new therapeutic strategy to overcome sorafenib resistance.

Several other mechanisms seem to be involved in the resistance to sorafenib in HCC (122) and Axl has been pointed out as having a regulatory role in this process. Increased mRNA and phosphorylation levels of several RTKs, including Axl, were reported in established sorafenib-resistant HCC cell lines (123). This aberrant Axl activation was confirmed by others, who also reported that Axl not only modulates motility and invasion capacities of sorafenib-resistant HCC cells, but also its downregulation increases the sensitivity of sorafenib-naïve and sorafenib-resistant cells to this drug (114). Corroborating these results, the authors retrospectively measured sAxl in serum from HCC patients treated with sorafenib and observed that higher sAxl levels were detected in patients who discontinued the therapy due to radiologically proven disease progression. More recently, Hsu and coworkers demonstrated that galectin-1 might also be associated with Axl signaling in the development of sorafenib-resistance in HCC cells. The authors observed that galectin-1 knockdown and overexpression were able to reduce and increase sorafenib resistance, respectively. In turn, both sorafenib-resistant HCC cells and HCC cells overexpressing galectin-1 exhibited increased Axl phosphorylation (124). Decreased chemoresistance to cisplatin was detected in human HCC MHCC-97L cells overexpressing the miRNA 34a-5p, a microRNA that directly targets Axl (125). Even though cisplatin is not conventionally used in HCC patients, this mechanism might have a broader relevance. Clinically, lower miRNA-34a-5p expression was associated with decreased overall survival in HCC patients. In recent years, other therapeutic agents were also approved to treat HCC. For instance, Cabozantinib, a multikinase inhibitor that has inhibitory activity against Axl and other RTK, was approved as second and third line therapy for the treatment of advanced HCC after failure of sorafenib (126).

Due to the lack of efficient biomarkers allowing early detection of HCC, most HCC cases remain undetected until they reach advanced stages. Therefore, soluble TAM receptors have been considered as candidate biomarkers for routine screening. In particular, sAxl serum levels were investigated in different multi- and single-center studies and significantly higher sAxl levels have been detected in HCC patients compared to healthy controls (101, 127, 128). Increased Axl protein levels in tumor tissue, evaluated by immunohistochemistry, were also associated with worse clinical prognosis and HCC recurrence (129).

## 3 Conclusions

TAM receptors play important roles in the progression of chronic liver disease. Their implication in processes such as inflammation, fibrosis, tumor cell growth and resistance to cancer therapy have gained interest in the last years, and the development of TAM-targeting strategies have shown to be useful in improving disease outcomes. In cancer research, TAM inhibitors proved to reduce proliferation and invasion of cancer cells, and Bemcentinib is currently in clinical trials for the treatment of different types of cancer. On the other hand, the role of TAM receptors in a MASLD context is still not fully understood and the impact of TAM modulators still needs to be further explored. For example, conflicting findings in the literature have been found regarding the mechanisms and therapeutic potential of MerTK in MASH. Although MerTK-targeting in experimental MASH models has demonstrated to reduce liver fibrosis, MerTK was also shown to confer hepatocyte protection against lipotoxicity through Gas6. Bemcentinib has been showing promising results in decreasing experimental MASH, implicating Axl as a potential therapeutic target for clinical practice. Several authors have suggested different new specific approaches such as pharmacological inhibitors, modulators and microRNAs that target TAM receptors, inhibiting liver fibrosis as well as proliferation, migration and invasion of HCC cells, both *in vitro* and *in vivo* models. Moreover, circulating TAM receptors represent promising biomarkers in fibrotic MASLD and HCC. Overall, we can conclude that TAM receptors represent interesting targets for the development of novel therapeutic approaches and for chemoprophylaxis of the progression of MASLD to cirrhosis and HCC.

## Author contributions

DA: Writing – original draft. LF: Writing – original draft, Writing – review & editing. FV: Writing – original draft. NV: Writing – original draft. RM: Writing – review & editing. FL: Writing – original draft. BF: Writing – original draft. MEB: Writing – review & editing. MP: Writing – review & editing. MB: Supervision, Writing – review & editing.
